# 2090 TL1 team approach to osteosarcoma cell detection

**DOI:** 10.1017/cts.2018.140

**Published:** 2018-11-21

**Authors:** Pablo J. Dopico, Henrietta Fasanya, Dietmar W. Siemann, Hugh Z. Fan

**Affiliations:** 1 Clinical and Translational Science Institute, University of Florida; 2 Department of Radiation Oncology, College of Medicine, University of Florida; 3 Department of Mechanical & Aerospace Engineering, Herbert Wertheim College of Engineering, University of Florida

## Abstract

OBJECTIVES/SPECIFIC AIMS: The objective of our collaboration is to develop a strong transdisciplinary team consisting of microfluidics engineers, cancer biologists, and clinicians, to identify cell surface markers capable of detecting circulating osteosarcoma cells (COC) using microfluidic devices. Our goals are 3-fold: (1) Identify cell surface markers unique to osteosarcoma (OS) for COC isolation, (2) develop a Geometrically Enhanced Mixing (GEM) device to isolate COCs, and (3) Evaluate the efficacy of GEM device to detect COCs in OS patients under treatment. The long-term goal is to utilize this cell detection approach to correlate the presence of COC with metastatic incidence. METHODS/STUDY POPULATION: To identify a marker to capture COCs we are utilizing flow cytometry and microfluidic capture devices. Flow cytometry will be used to evaluate the relative expression of epithelial cell adhesion molecule (EpCAM), CD45, cell surface vimentin (CSV), insulin-like growth factor 2 (IGF2R), interleukin 11 receptor subunit alpha (IL-11Rɑ), ganglioside 2 (GD2), and receptor activator of nuclear factor κ-B (RANK) on a panel of OS cell lines. These cell surface markers were selected based on an extensive review of OS cell surface markers. OS cell capture efficacy will be assessed by passaging a known concentration of OS cells through a GEM microfluidic device coated with antibodies targeting the selected marker, as indicated by flow cytometry. Once captured, COCs on the device will be analyzed and the capture efficiency for the indicated marker will be measured. ANOVA will be used to determine any significant difference in capture efficiency between marker types. Once an optimal marker or panel of markers has been selected we will conduct capture studies using OS cell spiked blood samples followed by clinical samples obtained from OS patients. In clinical samples, COC detection will be validated using the FDA approved triple immunocytochemistry technical definition of a circulating tumor cell (CTC). This will enable COCs to be differentiated from the normal whole blood cell population by selecting for CD45−, EpCAM+, and cytokeratin+ cells. RESULTS/ANTICIPATED RESULTS: Our preliminary studies have shown that on our microfluidic device, EpCAM, a marker commonly used to identify circulating tumor cells in other cancer settings, has a poor capture efficiency (15.9%+7.7%) for HU09 OS cells while the same setup with EpCAM has a capture efficiency of 56.9%+2.7% for BXPc-3 pancreatic cells. We therefore anticipate our flow cytometry studies to show a low expression of EpCAM and CD45 for OS cell lines, while showing a moderate to high expression of CSV, IGF2R, IL-11Rɑ, GD2, and RANK. We expect to show a 60%–80% capture efficiency for markers selected for COC capture. Currently, CSV and GD2 are particularly promising as markers based on previously published studies. DISCUSSION/SIGNIFICANCE OF IMPACT: OS is the most common primary bone tumor and the third leading cause of pediatric cancer deaths. At diagnosis 80% of patients will present with metastasis, however only 20% of these cases are clinically detectable. Innovative strategies to identify patients at risk of metastasis would allow for stratification of intervention therapies. Currently, tumor recurrence and metastasis are primarily dependent on diagnostic-imaging modalities such as computerized tomography or positron emission tomography scans. Unfortunately, these imaging modalities can only detect tumor masses of significant size (106 tumor cells). Liquid biopsies are a novel alternative to current diagnostic imaging systems to monitor metastatic incidence and treatment efficacy. The detection of CTCs through routine blood sampling has the potential to be used clinically for earlier detection, monitoring the treatment of metastatic cancers and surveying the effect of therapeutic interventions on metastasis. To date, the majority of the studies on CTCs have evaluated their presence in carcinomas. Although sarcomas are rare, they generally have a poor prognosis. This study will address one of the unmet medical needs in the field of CTC detection; the identification of cell surface OS makers to improve binding specificity, increase purity, and maintain a high capture efficiency. This phase of our proposal will evaluate the most abundant and conserved markers across a panel of OS cell lines. Once a marker or panel of markers is selected, we will begin to develop a microfluidic device that can be used clinically to detect CTCs in this disease setting.

